# Collectanea

**Published:** 1832-03

**Authors:** 


### COLLECTANEA.

**Floriferis ut apes in saltibus omnia libant,**
**Omnia nos, itidem, depascimur aurea dicta.**

### ANATOMY.

*Anomaly in the Pneumogastric Nerves.* M. Bignardi, professor of anatomy at Modena, has communicated to the Anatomical Society of Paris the dissection of a woman, in whom both pneumogastric nerves presented in their whole course a series of ganglions, some as large, others smaller, than the intervertebral ganglions of the spinal nerves, which they also resembled in structure. The great sympathetic on the left side was atrophied, and on this side the ganglions of the pneumogastric were both larger and more numerous. This woman during life exhibited nothing remarkable, except that she had a most voracious appetite.—*Rev. Méd.*

### PATHOLOGY.

*Case in which a large Needle was swallowed, and a year afterwards extracted from the Urinary Bladder, having formed the Nucleus of a Stone.* By — Logan, Surgeon, Lanark.

On the 29th January, 1830, Ann Wilson, aged twenty-two, a servant girl, of a robust habit and good consitution, residing in the village of Kilcadzow, three miles north of Lanark, having gone to bed with a large worsted needle in her mouth, swallowed it during her sleep. Next day, she had pain in the hypogastric region, when, recollecting to have had the needle in her mouth when she went to bed, and as she could nowhere find it, she attributed the pain to its presence in her stomach. Three days afterwards it had passed the pyloric orifice, and gained the small intestines, in which situation it seemed to have remained for a length of time, as, in May succeeding, when she first consulted me, she complained of permanent pain a little below the umbilicus on the left side: there was at this time no vesicular irritation, and she menstruated regularly until within two periods of the extraction of the needle.

I did not again see her till January 1831; but in the previous autumn she had complained of great pain in the situation of the bladder, accompanied with frequent calls to micturition, and passed a stone the size of a horsebean. In January 1831, the family attendant, Mr. Bouglas, of Carluke, sent her to me, desiring an opinion. By careful examination, I found that the point of the needle had entered the fundus of the bladder on the left side, and, being pushed on, had perforated the parietes of the opposite side, over the right acetabulum. The needle had formed a nucleus for the attachment of a calculus of considerable size, of a lobulated form, and having its larger extremity towards the orifice of the urethra. At this time she could not retain her urine above two or three minutes, which was passed with excessive pain, and was deeply tinged with blood, coagulating when cold. Introducing a pair of strong dressing forceps, I grasped the needle by a part free from any calculous incrustation, close to the mucous coat on the right side, and endeavoured, by pushing the whole backwards, to free the point, and bring it into the urethra; but this could not be effected by all the force which it was prudent to exert, on account of the long irritation having thickened the coats of the bladder, and produced such a diminution of its capacity as to grasp the calculus on all sides. Had this succeeded, the calculus might have been crushed, and the whole expelled without incision.

Being foiled in this attempt, we determined on cutting into the bladder, through the urethra. Eight leeches were applied over the pubis, and a gentle saline purgative administered. On the following day, the patient being secured in the usual manner, with the assistance of Mr. Bouglas, I passed a director into the bladder, previously injected, along which a slightly curved bistoury was run, making a lateral incision upwards, towards the left ascending ramus of the pubis. The finger of my right hand was then introduced, and, pushing gently but firmly upon the calculus, the point of the needle was disengaged by the left hand, and brought into the opening: the forceps were then applied, but, owing to the size of the calculus, it could not be extracted without enlarging the wound, which must have endangered the establishment of permanent incontinence of urine. The calculus, being of a friable nature, was crushed by the forceps, the needle withdrawn, and the fragments brought away by the scoop. The bladder was then washed out, an elastic gum-catheter introduced, and the patient put to bed. In the evening, twelve ounces of blood were taken from the arm, and leeches applied on the accession of any inflammatory symptoms. The bowels were kept open, and occasionally clysters administered, accompanied by warm fomentations. The wound suppurated on the eighth day. On the twenty-second day, she was able to sit by the fire and move about a little: she can now retain her urine two hours, and passes it by a voluntary inclination, offering a good prospect that there will be no permanent incontinence.

I am inclined to think the needle must have passed from the sigmoid flexure of the colon, as it had entered on that side of the fundus of the bladder. Had it come from the small intestines, it must have pierced the fundus in a more perpendicular direction. When the point had entered the parietes of the bladder, by the shortening of its muscular fibres, it must have been grasped and drawn in every time an action of the bladder was induced, aided by the peristaltic motion of the bowel acting on its head.—*Glasgow Med. Journal.*

---

*History of a Case in which, on Examination after Death, the Pancreas was found in a State of Active Inflammation.* By Wm. Lawrence, F.R.S.

Morbid changes of all kinds are extremely rare in the pancreas, as well as in the salivary and lacrymal glands, which are similarly organised. It seems doubtful whether the modern pathological anatomists, who have had the most extensive opportunities of observation, have ever seen inflammation of this gland: at least, no such affection is described by Baillie, Meckel, or Andral. "As for the pancreas," says the latter,* "I shall merely observe that its changes of structure are infinitely rare. I have found it redder than usual in some instances, and in others remarkably dense. In some bodies it has been compressed and diminished *(comme atrophié,)* by scirrhous or tubercular masses developed around it, or in the intervals of its lobules. On one

* Précis d'Anatomie Pathologique, tom. ii. p. 582.

occasion I found the hepatic extremity changed into a grayish white, hard, and homogeneous, substance, in which no trace of the normal organization could be observed. In another instance I found, in the middle of the gland, two small abscesses, each capable of holding a hazel-nut: but, in general, we may affirm that the pancreas is one of the organs most rarely diseased. To ascribe to it, therefore, an important part in certain gastric disorders, and to account for indigestion by changes in the pancreatic secretion, is purely hypothetical. The gland undergoes no observable change in the affections of the alimentary canal, or in those of the liver."

Dr. Baillie* met with one instance of abscess in the pancreas: the account of the symptoms, which was communicated to him by Dr. Heberden, is so short and imperfect as to render the case of little interest.

In his "Cours d'Anatomie Medicale,† Portal has a long catalogue of pancreatic diseases, including inflammation, suppuration, gangrene, scirrhus, cancer, and ulceration; and he seems to consider all of them as of common occurrence. This statement is quite at variance with the opinions of the authorities before mentioned, and with the result of my own experience. I can only explain the contradiction by supposing that M. Portal has adopted, with too little discrimination, the cases reported by older writers, and that he has admitted the existence of inflammation in the pancreas on insufficient grounds. He says that the pancreas has often been found inflamed, and that the stomach, duodenum, liver, spleen, and kidneys, have not been exempt from inflammation on the same occasions.

I offer the following case to the Society, because I have not met with any similar narrative in the course of my medical reading, and because it connects the symptoms and progress of the affection with the morbid changes which produced them.

CASE. I saw, in consultation with a physician and with the regular medical attendant of the family, a lady about twenty-one years of age, who had been delivered a few weeks previously of her first child. She had been very weak and excessively pale during the latter part of her pregnancy, and she became still more so after delivery. Her state and symptoms were like those of persons who have lost large quantities of blood; and her medical attendant considered that there was a defect in the process of sanguification. Under this view of the case, which was adopted by a physician who saw her soon after her confinement, cordials and stimuli, both medical and dietetic, were resorted to. No advantage resulted from this plan, and another physician was called in, who recommended calomel and opium, on the idea that inflammation had taken place in the chest, and that effusion had probably been the consequence. I saw her about thirty-six hours before death, when no hope of recovery could be entertained: she was excessively pale, with a rapid feeble pulse, hurried breathing, some fulness and uneasiness on the right side of the abdomen.

I learned afterwards that this lady had been most singularly troubled by thirst during her pregnancy, and that her mother, alarmed by her drinking cold fluids in large quantity, had represented to her that she feared the circumstance might prove injurious to the child. She had also suffered much from pain in the epigastric region, which was sometimes so severe as to oblige her to retire to her own apartment. In mentioning this circumstance, her mother drew her hand across the abdomen in the seat of her daughter's sufferings, and she pointed exactly to the situation of the pancreas. The gentleman who had regularly attended this lady was kind enough to favor me with the following account of her case from the commencement.

"My dear Sir: I have the pleasure of sending you the particulars of the symptoms which presented themselves in the case of Mrs. ——.

* Morbid Anatomy, chap. xii. † Tom. v. p. 351 et suiv.

"At the time Mrs. —— married, she appeared to be in good health. When she was between five and six months advanced in pregnancy, she lost her usual healthy appearance, and gradually became very pallid. This change I observed on occasionally meeting her in her walks from her own to her mother's house; and, on inquiring generally after her health, her answer invariably was, 'I am quite well.'

"About a month previous to her confinement, I, for the first time, was desired to see her professionally: she was then suffering from a severe attack of catarrh, accompanied with an incessant irritating cough; it was attended with very little fever, her pulse, in frequency, being but little above the natural standard. This complaint yielded in about ten days to the usual remedies, when she declared herself to be quite well, and during its continuance no symptom was complained of that was not strictly catarrhal. Her skin was then completely bleached, and the prolabia colourless. I did not again see her until the 29th of January, the morning on which her labour commenced; she then looked and felt extremely exhausted, and I was anxious as to the result of her labour. On making the usual inquiry, I found the presentation natural, the pains returning at pretty regular intervals, and she was delivered of a healthy female child. The placenta was expelled by the contraction of the uterus five minutes afterwards, and she did not, during the whole labour, lose two ounces of blood. The night after her labour was passed without pain; she was tolerably tranquil, but got little sleep. It was evident on the third day after her delivery, that, although the labour was comparatively easy, she had suffered much from the exertion. She felt so exhausted that she was constantly calling for sal volatile to smell, and occasionally to take internally, in order to prevent fainting; she sighed deeply and frequently. The least attempt to raise her head from the pillow produced a violent beating in the temples, but it subsided after a few minutes of perfect quietude. Her pulse was feeble and irritable, at about eighty-six beats in a minute; the bowels were rather relaxed; she was very thirsty, and had been so for three months previous to her delivery.

"On the fifth day after her confinement, Dr. —— saw her, and he repeated the examination I had previously made, by pressure with the hand over the whole abdominal cavity, in order to discover if there was tenderness in any part; but our patient declared most positively she felt neither pain nor soreness from the pressure. A similar examination was made some days afterwards, with the same result. The feeling of exhaustion continued to increase, but she never complained of pain, till about a week before her death, when, on pressing the abdomen, a slight uneasiness was felt about the situation of the caput coli. This was noticed the following day by Dr. ——, who directed a mustard poultice to be applied to the part. About five days previous to her death, the stomach became irritable, and nothing but rennet whey in small quantities was retained. She died exactly five weeks after her delivery.

"I should observe, that I do not recollect to have heard of Mrs. —— suffering severe pain in the epigastrium, till it was mentioned by her mother after her death: she certainly never named it to me herself, and it does appear somewhat extraordinary that, when it existed, it should not have been thought of sufficient consequence to call for medical assistance."

*Examination, thirteen hours after death.* The body had not lost its heat; the internal parts were warm to the touch.

The skin was universally and extremely pale.

No blood escaped on making the incisions necessary for exposing the abdomen and thorax, and for sawing round the skull.

The membranes lining the abdomen and thorax, and the viscera contained in those cavities, excepting the pancreas and spleen, were extremely pale, and almost bloodless. The appearance was like that observed in persons who have died of hemorrhage, or under the state described by the term anemia. The liver and kidneys were pale, and the several portions of the alimentary canal quite white, without any traces of blood in them.

The heart was pale, and rather large; its cavities and the contiguous large vessels contained some fluid of watery consistence, about the colour of red wine, and small portions of soft coagula. The coronary vessels contained no blood. The muscular substance of the heart was pale, and rather flaccid; the structure of the organ in other respects was natural. The lungs were healthy, except that frothy fluid escaped on cutting into their posterior part. The cellular texture around the pancreas and duodenum, the great and small omentum, the root of the mesentery, the mesocolon, and the appendices epiploicæ of the arch of the colon, were loaded with serous effusion. The fluid, which was transparent, bright yellow, and of watery consistence, ran out in large quantity on cutting into the parts above mentioned, which were distended in some places to the thickness of two or three inches.

The pancreas was throughout of a deep and dull red colour, which contrasted very remarkably with the bloodless condition of other parts. It was firm to the feel externally, and, when an incision was made into it, the divided lobules felt particularly firm and crisp; the texture was otherwise healthy. The part was left wrapped up in a cloth for nearly forty-eight hours after its removal from the body, the weather being then very cold. At the end of this time the hardness was gone, and the gland appeared rather soft.

The spleen was rather large and turgid, livid externally, brownish red internally, and somewhat soft in texture.

The surface of the dura mater, covering the cerebral hemispheres, was lined, in the neighbourhood of the falx, with a very thin, soft, and almost mucilaginous, layer of light-red tint; it could be scraped off with the handle of the knife, leaving the membrane of its natural appearance. There was slight serous infiltration of the pia mater. The blood-vessels of the brain were moderately full. The distention of the cellular membrane by serous effusion in this instance was analogous to the œdematous swelling which often occurs round other parts when actively inflamed.

The pancreas is not unfrequently found after death, as it was in this case, preternaturally hard; and I suppose that the gland has been in this state in the numerous instances in which we hear and read of its having been scirrhous. Although I do not know on what this hardness depends, I have never considered it as a morbid condition; because it occurs in individuals who have died of other diseases, without any symptoms referable to the pancreas; because the structure of the part is perfectly healthy in all other respects; and because the hardness soon disappears after death, as it did on this occasion.—*Medico-Chir. Transactions*, vol. xvi.

---

MISCELLANEOUS.

*Observations on Pulmonary Hemorrhage.* Taken from a Clinical Lecture delivered at the Middlesex Hospital, by Dr. Watson.

Gentlemen: Two deaths have occurred among my patients during the last week. They both were the result of the same disease, and present some points worthy of notice.

Elizabeth L——n, aged forty-one, a cook-maid, was admitted on the 8th of November, for an herpetic eruption, of which I shall not detain you by saying any thing more than it rapidly got better, and had, before her death, nearly disappeared, under the use of the zinc ointment.

It was soon discovered, however, that she laboured under a disease of a much more formidable kind. She complained, on her admission, of some pain in the left side of the chest, of pain and tenderness at the pit of the stomach, and of cough; and stated that she expectorated daily a considerable quantity of thich yellow phlegm, but that she had never spat blood. The cough had existed, more or less, for the last nine months; i. e. from the time when she had had an attack of "inflammation on the chest." During this period she had been much more liable than before to "catch cold."

Now these symptoms were by no means distinctive. They might all have been explained upon several different suppositions. She might have had pneumonia at the period she spoke of; or pleurisy; or inflammation; or some other morbid condition of the heart or pericardium; any of which might have led to changes in the organs of respiration calculated to produce the existing symptoms; or she might have been labouring all along under bronchitis; or the symptoms might be the result of tubercular disease of the lungs.

I may observe here, that she had not a phthisical look, and was not remarkably thin.

When I first had an opportunity of examining the matter expectorated, I saw much reason to fear that the last and least favorable of the suppositions just mentioned was the fact, the true one. The expectoration consisted of globular, yellowish, separate masses, of peculiar appearance, floating in a thinner watery fluid. Sputa of this kind are strongly characteristic of phthisis pulmonalis; still they are not conclusive of its existence. The application of the stethoscope to the patient's chest removed all doubt about the nature and stage of the disease completely, and in less than a minute.

Below the left collar-bone, and close to the shoulder-joint, the air respired by the patient sounded as if it were blown through a hollow tube of some size, close to the ear of the listener. The voice also, loud and harsh, seemed to pass along the instrument. Neither of these peculiar sounds were heard, nor any morbid sound, in the corresponding portion of the right side of the chest. There was unquestionable *pectoriloquy* on the left side. I could no longer doubt that a tubercular cavity or cavities existed in the upper part of the left lung, and that consequently our patient (without at all suspecting it herself) bore about her a disease which must in no long time prove fatal to her.

She remained in the hospital till December the 3d. On that day, as the skin was nearly well, and the pectoral symptoms appeared stationary, it was arranged at the visit that she should leave the house on the following Tuesday. In the afternoon of the same day, during a violent fit of coughing, she brought up suddenly a large quantity of blood from the lungs, and presently expired.

The left lung, in its upper lobe especially, was found, as you know, full of tubercles. There were several cavities near its summit, communicating with each other and with the larger air-tubes. A thin string of blood was traceable into one of these, the most remote from the trachea, where it ended in a small irregular clot. No vessels from which the blood might have proceeded were detectible. We once fancied, indeed, that we saw the orifice of a minute blood-vessel, but we could not satisfy ourselves that it was so; if it were, it must have been an extremely small one.

There were few or no marks of disease in the other lung.

In this case, you will do well to remark the facility with which the diagnosis was determined by auscultation, the occurrence of death during the first attack of hemorrhage, and the absence of any rupture of a large vessel to explain that hemorrhage.

The other patient to whom I have alluded was Daniel E——n, a stableman, fifty-seven years old. He came into the hospital on the 22d November, and died December 8th.

He complained of cough, which harassed him at night especially, or whenever he lay down. He said he had had a bad cough about three weeks; that he expectorated white phlegm; that, about a fortnight before, he had coughed up suddenly, and for the first time, nearly a pint of dark-coloured blood, and that he had spat blood in smaller quantities several times since.

He admitted that he had been subject to cough, every winter, for years; but it had never before "laid him up." He stated that he had lately been obliged to sit up in bed for three nights successively. We always found him lying upon his right side, and he told us that any other position immediately increased his cough. As you saw these patients from day to day, I only remind you of the most prominent of their symptoms. You know that auscultation gave precisely the same results, the same unequivocal evidence of pectoriloquy, as in the former case; only here it was in the right lung.

Now this man was not emaciated, and bore in his countenance none of the characters of the disease of which he was dying.

You saw the result of the post-mortem examination. The right lung was loaded with tuberculous matter, and had a considerable cavity in its lobe. The left was comparatively sound. The cavity communicated with one of the principal bronchial divisions, by means of a large straight air-tube running obliquely from the cavity downwards and inwards. The circumstance explains very satisfactorily the effect of position in emptying the cavity and exciting cough.

Here, again, you will notice the copious hæmoptysis occurring, for the first time, at an advanced period of the disease; and the decisive information furnished by a single application of the stethoscope, in regard to the real nature (of which we might otherwise have been uncertain) of our patient's complaint. There are now four or five other instances of the very same thing amongst my own patients in our wards.

I shall take the opportunity afforded me by these two cases, and by another case which has also terminated fatally here during the last week, and of which I shall speak presently, to offer you some remarks upon pulmonary hemorrhage; chiefly in respect to its pathology, and its general import as a prognostic symptom. You ought to know, that "spitting of blood" may depend upon several distinct morbid conditions; it will be my object to explain some of these conditions, and, if I can, to render your future observation of this symptom, whether here or elsewhere, more interesting and more instructive than it might otherwise be.

It is commonly said of a person who happens to spit blood, that he has broken a blood-vessel in his lungs. When blood flows profusely from an external wound, you may often see that it issues from a large vessel which has been laid open; and hence, by a very natural though deceptive analogy, you might be led to infer, that, in every copious hemorrhage, whether from an external wound or from internal parts through some of the natural outlets of the body, the blood really escapes through an opening in the sides of some one (or more) considerable blood-vessel. This used to be held as good medical doctrine; but the progress of pathological knowledge has shewn it to be unsound. It is still the doctrine of the public, and it even continues to be sanctioned by the language and writings of many of the members of our own profession.

It is, indeed, quite true that some cases of pulmonary hemorrhage do depend upon the rupture of vessels of a certain magnitude; but it is no less true that, in by far the greater number of instances, there is no lesion, detectible by dissection, of either veins or arteries; but the blood is poured out by (what is called) *exhalation*; i. e. it proceeds from those natural pores or channels which constitute, or are connected with, the ultimate ramifications of the minuter blood-vessels, and which are called exhalants. This is true, not only of bleeding from the lungs, but of internal hemorrhage generally, with the exception, perhaps, of cerebral hemorrhage.

The particular vessels injured in the first and smaller class of cases, and the nature and cause of the breach made in their sides, are matters subject to infinite variety. Sometimes the apertures through which the blood is extravasated are the results of a disorganizing process which has commenced in the coats of the vessels themselves; as when, for example, large aneurisms of the thoracic aorta, or of its primary branches, burst and pour its contents into the tracheal or bronchial tubes. Sometimes a large blood-vessel is laid open by the gradual encroachment and extension of disease from contiguous parts.

It would be very natural for you to suppose, that where the hemorrhage (as in the two cases before us) has been copious, and co-existent with tubercular excavations in the lung, the blood has proceeded from large vessels which had been laid open during the softening and expulsion of the tubercles, or by the subsequent extension of the ulcerating cavities. But, in fact, this is very rarely the case; and some modern writers have sought to explain the infrequency of hæmoptysis from this cause, by stating that the arterial tissue possesses a low degree of vitality, and thereby resists and avoids the destruction in which the surrounding tissues are involved. This explanation supposes that the bands which are not unfrequently seen stretching across tubercular vomicæ, are really blood-vessels that have escaped the disorganizing process. Such seems to have been the notion entertained by Bayle, and it has been more recently and more expressly advanced by Cruveilhier. But according to the more accurate observation of Laennec and Andral, the bands observed, in some cases, thus crossing the cavities, are composed of the natural tissue of the lung; condensed, indeed, and often loaded with tuberculous matter, but seldom containing blood-vessels. Laennec states expressly, that "he never found a vessel of any consequence included within the substance of these bands, which he resembles to the columnæ carneæ of the heart in appearance; that no such example occurs in Bayle's book, who had, however, mentioned to him, in conversation, one instance only in which fatal hemorrhage took place from the rupture of a vessel that extended across a very large cavity." In the few cases in which Laennec had been able to detect blood-vessels at all in these bands, they formed but a small part of their thickness, were generally obliterated, and traceable for a very small distance only; the vascular appearance being soon lost in the pulmonary tissue of which the columns consisted. According to the same author, the tubercles press aside and separate the blood-vessels, which are often to be seen, of considerable size, lining, as it were, and forming a part of, the sides of a cavity; sometimes a little flattened, but seldom obliterated, while their smaller ramifications, which extend towards the excavations or towards crude tubercles, are become quite impervious. Dr. Baillie had in this country noticed previously the same condition of these vessels. "When blood-vessels (he says) are traced into an abscess of the lungs, I have found them, upon examination, very much contracted just before they reach the abscess, so that the opening of their extremities has been closed up entirely. On such occasions it will require a probe to be pushed with a good deal of force, in order to open again their extremities. In these contracted vessels the blood is coagulated, as it is under similar circumstances in other parts of the body. This change in the blood-vessels is, no doubt, with a view to prevent large hemorrhages from taking place, which would certainly be almost immediately fatal."

Andral, again, states that he had only once seen the orifice of a ruptured vessel from which the blood that filled a tubercular cavity had probably proceeded. This vessel was contained in one of the bands that had traversed the empty space, and had been broken. The mouth of the vessel was plugged by a small fibrinous coagulum, which was easily extracted, and then the pervious condition of the vessel became apparent.

Occasionally, but rarely, the cavities of which I have been speaking are found full of blood, though no lacerated vessel can be traced in them. More commonly the tuberculous or puriform matter which they contain is more or less tinged of a red colour, from the admixture of blood. In both these cases the blood seems to be the result of a sort of exhalation from the sides of the cavity.

All the examples of bleeding from the lungs that I have hitherto mentioned, may be looked upon as illustrations of the primary species of symptomatic hemorrhage; of hemorrhage, i. e. symptomatic of organic disease in the very part from which the blood proceeds.

But in a far greater number of cases the blood, in hæmoptysis, is exhaled from the mucous membrane that lines the air-passage; for, when this surface is examined in the dead body, and immediately after the occurrence of pulmonary hemorrhage, it is often found to be perfectly entire, from the commencement of the trachea to the remotest divisions of the bronchial tubes; as far, at least, as minute dissection can follow them. The membrane in such cases is sometimes partially red; sometimes pale, or with scarcely any trace of vascularity. The former of these appearances results from the continued turgescence of the capillary vessels; the latter is the consequence of their having been completely emptied by the ultimate hemorrhage. All this is in strict analogy with what is well known to occur in hemorrhages from other mucous surfaces, and especially from that of the alimentary canal.

When the blood is thus exhaled from the mucous membrane of the air-passages, the hemorrhage may be purely idiopathic, i. e. it may be independent of any discoverable alteration of texture, either in the mucous surface itself, or in any other part which, by reason of some intelligible connexion of structure or relation, seems capable of influencing the capillary circulation of that membrane. But the occurrence of pulmonary hemorrhage strictly idiopathic has probably been more frequently affirmed than proved. Active hemorrhage from the lungs is stated by systematic writers to be the hemorrhage of adolescence, as epistaxis is that of childhood. I believe, however, that idiopathic hæmoptysis is very rare indeed, unless we may consider as such certain cases of vicarious hemorrhage, which are sufficiently common. Andral informs us, that in one instance only, in which hemorrhage from the surface of the air-passages had been the immediate and apparently the sole cause of death, had he ever found the substance of the lungs free from tubercles and perfectly healthy. He does not, however, state wheher in this instance the heart also was in its natural condition; an important omission, as I shall presently shew you. He relates, indeed, as an example of idiopathic hæmoptysis, the case of a young man who suffered profuse hemorrhage from the lungs on four several occasions, between the age of twelve and eighteen, without any apparent detriment to his health, which remained excellent. It is consistent, however, with much experience, to suppose that crude tubercles might have occupied the lungs in this person, and have sufficed, on the application of some exciting cause, to determine the hemorrhage, although as yet their presence was not indicated by any other sign. Almost every systematic writer quotes, as an example of idiopathic hemorrhage from the lungs, the story of a Roman governor (mentioned by Pliny) who lived to the age of ninety, though he was afflicted with habitual hæmoptysis. The frequent citation of this supposed instance is of itself a sufficient proof that spontaneous pulmonary hemorrhage is far from being common.

*Cæteris paribus*, the disposition to pulmonary hemorrhage is increased by whatever tends to diminish the capacity of the thorax, and to compress the lungs. And it is on this principle that some have attempted to account for the frequency of hæmoptysis in persons with crooked spines; in tailors who sit continually in a bending posture; in young women who apply tight lacing round the lower part of the chest; and in those who labour under dropsy of the belly. Hæmoptysis accompanying ascites has been known to cease at once on the performance of the operation of tapping, and to recur upon the re-accumulation of the dropsical fluid; and this not on one occasion only, but so often and regularly as to preclude all notion of accidental coincidence. There can be little doubt, however, that, in this class of cases, or at least in a vast majority of them, the hæmoptysis is mainly to be ascribed to organic disease of the heart or of the lungs.

There is a form of pulmonary hemorrhage, to which I have already alluded as being common; which is sometimes called constitutional, and which is vicarious of some other natural or morbid discharge, and most frequently of all of the menstrual discharge in females. If this cannot strictly be regarded as idiopathic, it may be considered as forming a link of connexion, as lying midway, between symptomatic and idiopathic hemorrhages. Some remarkable examples of this kind of hæmoptysis have fallen under my observation in this hospital. It is sometimes, but rarely, attended with danger to life. There are many very curious and well-authenticated facts upon record concerning this singular form of hemorrhage by deviation, as it is sometimes called. Women have been known to menstruate in this way, if the expression be allowable, through the lungs, throughout nearly the whole of that portion of life during which that function usually continues; and this without any impairment of their general health. You will find such cases detailed by Pinel and by Hoffman. At present I can only thus briefly allude to them.

It is, however, a melancholy fact, that the hemorrhage which takes place by exhalation from the mucous membrane of the air-passages, is dependent, in a very large proportion of instances, upon incurable organic disease. Such cases belong to the secondary species of the symptomatic hemorrhage; the disease of which it is symptomatic being seated elsewhere than in the very membrane whence the blood escapes. The complaint of which hæmoptysis is by far the most frequently symptomatic, is undoubtedly tubercular phthisis. Where the tubercles are found upon dissection to be yet crude and entire, and no breach can be detected in the mucous membrane, no question can be entertained about the immediate seat of the bleeding; and even where cavities exist, especially if they contain no blood, it is probable that in most cases the hemorrhage has had a similar origin.

When hæmoptysis is thus actually symptomatic of tubercular disease of the lungs, it is liable to considerable variety in regard to the period of its first occurrence, and the symptoms by which it is succeeded.

There are many persons in whom the first attack of hæmoptysis precedes, for months, and even for many years, the primary symptoms of unequivocal phthisis.

There are others in whom the first attack of hæmoptysis is immediately followed by all the well-known signs which declare the existence of tubercles in the lungs.

Many, again, do not spit blood until the tubercles have reached a considerable degree of advancement, and the phthisical symptoms have been for some time clearly marked; and occasionally in such cases, as in the instance of our female patient, the first hemorrhage proves fatal.

Lastly, it is far from being an uncommon thing to see pulmonary consumption run its whole course, and terminate in death, without having been attended with any expectoration of blood.

Andral gives the following statement as the result of his own observation, in regard to the relative frequency of these several modes of connexion between hæmoptysis and consumption:

Of the persons whom he had known to die of this disease, one in six never spat blood at all. Three in six, or one half of the whole number, did not spit blood until the existence of tubercles in the lungs was already made certain by unequivocal symptoms: in the remaining two sixths the hæmoptysis preceded the other symptoms of tubercular disease, and seemed to mark the period of its commencement.

By this comparative statement you will see how very frequently hæmoptysis occurs as one of the symptoms connected with tubercular phthisis. Under this author's observation it happened in five cases out of six. In Louis's experience, however, the proportion, though very large, is not quite so great as Andral found it. Among eighty-seven instances of consumption there were fifty-seven, or four in every six, in which hæmoptysis had been present.

You may perhaps wonder that I so frequently refer to these French authorities. I do so for two reasons; 1st, because I wish to excite your attention to their writings, which (those of Andral and Laennec especially) are full of valuable and interesting matter; and 2dly, because they supply me with what is much wanted among ourselves, viz. conclusions drawn from trustworthy numerical statements made on a large scale. The practical information to be derived from tabular accounts of this kind is perhaps more useful, as it is certainly more secure from fallacy and suspicion, than almost any other.—*Med. Gazette.*

